# Optimization of (Dithioperoxo)thiolate-Based Antifungal Agents for Triazole-Resistant *Aspergillus Fumigatus*

**DOI:** 10.3390/pathogens14090878

**Published:** 2025-09-03

**Authors:** Surya Karuturi, Kaitlyn L. Jobe, Melinda E. Varney, Michael D. Hambuchen, A. R. M. Ruhul Amin, Timothy E. Long

**Affiliations:** Department of Pharmaceutical Sciences, School of Pharmacy, Marshall University, Huntington, WV 25755, USA; karuturi@marshall.edu (S.K.); kaitlyn.jobe99@gmail.com (K.L.J.); varney31@marshall.edu (M.E.V.); hambuchen@marshall.edu (M.D.H.); amina@marshall.edu (A.R.M.R.A.)

**Keywords:** antimicrobial discovery, antifungal, *Aspergillus*, disulfides

## Abstract

This investigation on novel antifungal agents featuring a thiol-reactive (dithioperoxo)thiolate chemical nucleus [-NC(S)S-SR] established that the optimal levels of fungal growth inhibition were achieved with thiomethyl-bound derivatives (R = Me). The most efficacious analogs had MIC_50_/MIC_90_ values of 2/2 µg/mL and an MIC range of 1 to 2 µg/mL for a ten-member panel of voriconazole-resistant *A. fumigatus* mutants. Pharmacodynamic studies revealed that the lead (dithioperoxo)thiolates impaired conidial germination and germling development more effectively than voriconazole for the triazole-resistant strain AR-1295. Moreover, glutathione and Cu^2+^ were shown to have antagonistic interactions, which was attributed to the thiol-reactive, pro-oxidant properties of the (dithioperoxo)thiolates and their metabolic conversion to chelating agents. Cytotoxicity studies further showed that the compounds were less toxic to human fetal kidney cells than squamous carcinoma cells. The collective findings of the investigation indicate that (dithioperoxo)thiolates are effective antifungal agents against *A. fumigatus* to merit additional research on their therapeutic potential.

## 1. Introduction

*Aspergillus* is a genus of conidium-forming filamentous molds that can infiltrate the respiratory tract to spawn invasive mycoses in individuals with deficient fungal immunity [1]. Multiple species of *Aspergillus* are implicated in aspergillosis, with *A. fumigatus* being the most common clinical isolate in pulmonary and disseminated infections [2]. Oncology and transplant patients, including hematopoietic stem cell recipients requiring immunosuppressive therapy (e.g., sirolimus), are at greatest risk for disseminated invasive aspergillosis [3]. The primary treatment and prophylaxis for *A. fumigatus* infections are the triazole antifungals voriconazole, posaconazole, and isavuconazole [3,4]. The Clinical and Laboratory Standards Institute (CLSI) and European Committee on Antimicrobial Susceptibility (EUCAST) have established a resistance breakpoint of ≥2 µg/mL for first-line voriconazole following international surveillance reports that indicated 97.8% of clinical isolates exhibited a MIC of 1 µg/mL or less [5]. However, experts predict up to 15% of patients receiving voriconazole or comparable triazole therapy will require an alternative treatment due to drug intolerance or resistance [6].

To this end, the agricultural use of azole fungicides has largely contributed to the emergence of triazole-resistant *A. fumigatus*. Resistance is associated with point mutations in the *cyp51A* gene that weakens the binding of triazole antifungals to the encoded protein target, lanosterol 14α-demethylase [7]. Triazole resistance may also be conferred by the overexpression of efflux transporter genes (e.g., *cdr1B*) in *A. fumigatus* [8]. Currently, there are few alternative drugs when these most relied on antifungal therapies are deemed ineffective or contraindicated due to toxicity or an interaction with other cyp3A substrates (e.g., sirolimus). In the event of triazole treatment failure, clinicians will resort to a six- to twelve-week course or longer of liposomal amphotericin B [3]. Salvage therapy with caspofungin or micafungin may then be used for refractory infections [3]. Thus, there is a critical need to develop additional second-line antifungal drugs when triazole-based therapies cannot be administered to aspergillosis patients.

**Figure 1:**
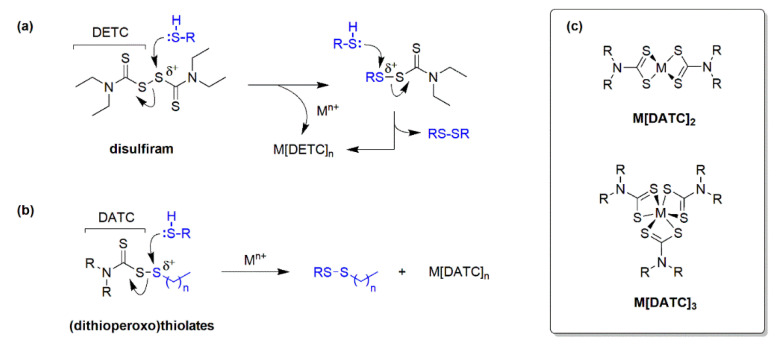
Thiol-disulfide exchange reactions of thiols (RSH) with disulfiram and (dithioperoxo)thiolates. (**a**) Reactions between disulfiram and RSH generates *N*,*N*-diethyldithiocarbamate (DETC) that can form M[DETC]_n_ complexes with metal ions (M^n+^). (**b**) Reactions between (dithioperoxo)thiolates and RSH generates *N*,*N*-dialkyldithiocarbamates (DATCs) that can form M[DATC]_n_ complexes with M^n+^. (**c**) Chemical structures of M[DATC]_n_ complexes containing divalent and trivalent metal ions.

Drug repurposing and repositioning are effective strategies to develop a new treatment from a pre-existing medication with established human pharmacokinetic and safety data. In a recent report, Shanholtzer and coworkers disclosed that fluconazole-resistant *Candida auris* and *Candida glabrata* were susceptible to the alcohol sobriety aid disulfiram (Antabuse^®^) [9]. Moreover, the yeasts were moderately sensitive to the primary metabolite of disulfiram, diethyldithiocarbamate (DETC). Figure 1a depicts the thiol-disulfide exchange between disulfiram and a reducing thiol (e.g., glutathione, cysteine) that generates DETC. Upon release, the metabolite is believed to sequester metal ions (M^n+^) such as Zn^2+^ and Fe^3+^ as a secondary mechanism of microbial growth inhibition [10,11,12]. Interestingly, co-treatment with Cu^2+^ salts was shown to decrease the minimal inhibitory concentrations (MICs) of disulfiram and DETC against *Candida* species but at the cost of reduced fungicidal activity that was attributed to the formation of Cu[DETC]_2_ [9].

The findings of this research prompted us to evaluate (dithioperoxo)thiolate analogs of disulfiram (Figure 1b) as antifungal agents. We previously reported that (dithioperoxo)thiolates had an optimal MIC range of 0.25 to 1 µg/mL for *Candida albicans* compared to 2 µg/mL for disulfiram [13]. The structure–activity relationship (SAR) analysis indicated that analogs with short *S*-alkyl chains were the most efficacious for antifungal activity. The analysis further revealed that *N*,*N*-dimethyl and *N*,*N*-diethyl substituents conferred the lowest MICs for *C. albicans*. The primary objective of this investigation was to identify lead (dithioperoxo)thiolates with optimal antifungal activity against triazole-resistant *A. fumigatus*. In addition, the in vitro antifungal pharmacodynamics and cytotoxic activity were assessed for compounds with optimal MIC_50_/MIC_90_ values.

## 2. Materials and Methods

### 2.1. Synthesis

Chemicals were obtained from Thermo Fisher Scientific (Waltham, MA, USA) or VWR international (Radnor, PA, USA) and used without further purification. Reactions were monitored by thin-layer chromatography (TLC) using GF_254_ silica-gel-coated aluminum TLC plates. Compounds were purified using a Biotage^®^ Isolera™ chromatography system (Charlotte, NC, USA) with KP-Sil silica gel columns and elution detection set at 254 nm. ^1^H and ^13^C NMR data were recorded on a Bruker AVANCE™ III HD 300 MHz spectrometer (Bruker, Billerica, MA, USA) and reported as δ values in ppm relative to tetramethylsilane (TMS). Coupling constants were recorded in Hz, and multiplicity reported as follows: s (singlet); bs (broad singlet); d (doublet); t (triplet); q (quartet); m (multiplet); dd (doublet of doublets); dt (doublet of triplets); dq (doublet of quartets); and tt (triplet of triplets). Mass spectra were recorded on an Agilent 5977B GCMS (Agilent, Santa Clara, CA, USA).

#### 2.1.1. Preparation of Carbamo(dithioperoxo)thiolates (**1a–d**); General Procedure

Disulfiram (200 mg, 0.68 mmol) and the appropriate mercaptan (0.74 mmol) were reacted in a seal tube at 60 °C in 2 mL of anhydrous dimethylformamide (DMF) [13,14]. After stirring for 18–24 h, the mixture was cooled to room temperature, diluted with water, and extracted twice with hexanes (2 × 3 mL). The organic layers were combined, washed with water (3 × 2 mL), dried over MgSO_4_, filtered, and concentrated in vacuo. Silica gel chromatography with 0–10% EtOAc in hexanes provided the pure carbamo(dithioperoxo)thiolates whose characterization data matched that previously reported [13,14].

#### 2.1.2. Preparation of *N*-Alkylthiophthalimide (**2**); General Procedure

Sulfuryl chloride (2.4 g, 18 mmol) in 5 mL of CH_2_Cl_2_ was slowly added dropwise to a stirring solution of methyldisulfide or ethanethiol (18 mmol) in 20 mL of CH_2_Cl_2_ chilled in an ice bath [15]. Upon completion, the ice bath was removed, and the mixture was stirred for 30 min. The solution was transferred to an addition funnel and added dropwise to an ice-chilled suspension of phthalimide (2.4 g, 16.4 mol) in 25 mL CH_2_Cl_2_ containing triethylamine (2.2 g, 21.3 mmol). Upon completion, the ice bath was removed and the mixture stirred for 1 h. The solution was then washed twice with deionized water, dried over MgSO_4_, filtered and evaporated. Recrystallization from boiling hexanes provided the pure *N*-alkylthiophthalimides whose characterization data matched that previously reported [15,16].

*N*-methylthiophthalimide (**2a**): white solid (36%), m.p. 178–179 °C; TLC (SiO_2_) *R*_f_ 0.38 (hexanes:EtOAc 3:1); ^1^H NMR (300 MHz, CDCl_3_) δ 8.02–7.72 (m, 4 H), 2.55 (s, 3 H); ^13^C NMR (75 MHz, CDCl_3_) δ 168.3, 134.7, 132.3, 124.0, 22.6; MS (EI, 70 eV): *m*/*z* (%) = 193 (22) [M], 160 (23), 148 (52), 130 (100), 104 (26).

*N*-ethylthiophthalimide (**2b**): white solid (70%), m.p. 115–117 °C; TLC (SiO_2_) *R*_f_ 0.45 (hexanes:EtOAc 3:1); ^1^H NMR (300 MHz, CDCl_3_) δ 7.94 (m, 2 H), 7.80 (m, 2 H), 2.93 (dq, *J* = 13.3, 7.6, 6.5 Hz, 2 H), 1.29 (dq, *J* = 12.9, 7.0, 5.9 Hz, 3 H); ^13^C NMR (75 MHz, CDCl_3_) δ 13C NMR (75 MHz, CDCl3) δ 168.6, 134.6, 132.1, 123.9, 32.7, 13.2; MS (EI, 70 eV): *m*/*z* (%) = 207 (5) [M], 148 (100), 130 (100), 104 (40).

#### 2.1.3. Preparation of Carbamo(dithioperoxo)thiolates (**1e–i**); General Procedure

Carbon disulfide (60 µL, 1.1 mmol) was added dropwise to a stirring solution of amine (0.9 mmol) and KOH powder (51 mg, 1 mmol) in diethyl ether (2 mL) at 0 °C. After 1 h, the solvent was evaporated under reduced pressure. The K^+^ salt was filtered, washed with ether, and resuspended in benzene (5 mL) containing *N*-(alkylthio)phthalimide (0.9 mmol). After stirring for 2 h, hexanes was added, the salts were removed by filtration and the filtrate was evaporated. Silica gel chromatography with 0–10% EtOAc in hexanes provided the pure carbamo (dithioperoxo)thiolates whose characterization data matched that previously reported [13,14].

methyl dimethylcarbamo(dithioperoxo)thioate (**1e**): pale oil (48%); TLC (SiO_2_) *R*_f_ 0.37 (hexanes:EtOAc 9:1); ^1^H NMR (300 MHz, CDCl_3_) δ 3.60 (s, 3 H), 3.45 (s, 3 H), 2.51 (s, 3 H); ^13^C NMR (75 MHz, CDCl_3_): δ 197.0, 47.1, 41.5, 22.3; MS (EI, 70 eV): *m*/*z* (%) = 167 (100) [M], 135 (33), 123 (16), 121 (11), 120 (100).

methyl diethylcarbamo(dithioperoxo)thioate (**1f**): pale oil (42%); TLC (SiO_2_) *R*_f_ 0.63 (hexanes:EtOAc 9:1); ^1^H NMR (300 MHz, CDCl_3_) δ 4.06 (h, *J* = 6.7 Hz, 2 H), 3.79 (tt, *J* = 12.5, 6.9 Hz, 2 H), 2.50 (s, 3 H), 1.32 (d, *J* = 7.9 Hz, 6 H); ^13^C NMR (75 MHz, CDCl_3_) δ 195.5, 51.5, 46.9, 22.9, 13.0, 11.5; MS (EI, 70 eV): *m*/*z* (%) = 195 (4) [M], 116 (100).

ethyl dimethylcarbamo(dithioperoxo)thioate (**1g**): oil; ^1^H NMR (300 MHz, CDCl_3_) δ 3.64 (s, 3 H), 3.51 (s, 3 H), 2.99–2.82 (m, 2 H), 1.35 (t, *J* = 7.4 Hz, 3 H); ^13^C NMR (75 MHz, CDCl_3_) δ 197.5, 47.2, 41.6, 32.2, 13.7; MS (EI, 70 eV): *m/z* = 181 [M].

ethyl benzyl(methyl)carbamo(dithioperoxo)thioate (**1h**): oil; rotamers observed: ^1^H NMR (300 MHz, CDCl_3_) δ 7.52–7.05 (m, 5 H), 5.38 (s, 1 H), 5.07 (s, 1 H), 3.50 (s, 1 H), 3.43–3.25 (m, 2 H), 3.06–2.73 (m, 2 H), 1.40–1.14 (m, 3 H); ^13^C NMR (75 MHz, CDCl_3_) δ 199.3, 198.1, 135.1, 134.4, 129.0, 128.8, 128.1, 127.9, 127.8, 127.1, 61.6, 57.9, 45.1, 38.8, 32.3, 13.7; MS (EI, 70 eV): *m/z* = 257 [M].

methyl methoxy(methyl)carbamo(dithioperoxo)thioate (**1i**): pail oil (39%); TLC (SiO_2_) *R*_f_ 0.42 (hexanes:EtOAc 9:1); ^1^H NMR (300 MHz, CDCl_3_) δ 3.84 (s, 3 H), 3.75 (s, 3 H), 2.44 (s, 3 H); ^13^C NMR (75 MHz, CDCl_3_) δ 194.9, 61.6, 40.8, 22.5; MS (EI, 70 eV): *m*/*z* (%) = 183 (31) [M], 126 (30), 104 (100).

#### 2.1.4. Preparation of Carbano(dithioperoxo)thioates (**3**); General Procedure

Carbon disulfide (54 µL, 0.9 mmol) was added dropwise to a stirring solution of alkyl alcohol (0.9 mmol) and KOH powder (51 mg, 1 mmol) in diethyl ether (5 mL) at 0 °C. After 1 h, the solvent was evaporated under reduced pressure. The K^+^ salt was washed with ether, filtered, and resuspended in benzene (5 mL) containing *N*-(alkylthio)phthalimide (0.9 mmol). The mixture was stirred at room temperature until the reaction was complete. Hexanes was then added, the salts were removed by filtration, and the filtrate was evaporated. Silica gel chromatography with 0–10% EtOAc in hexanes provided the pure carbano(dithioperoxo)thioates whose characterization data matched that previously reported [17,18].

(methoxy(thiocarbonyl))methyldisulfane (**3a**): pale oil (46%); TLC (SiO_2_) *R*_f_ 0.69 (hexanes:EtOAc 9:1); ^1^H NMR (300 MHz, CDCl_3_) δ 4.28 (dd, *J* = 6.1, 3.0 Hz, 5 H), 2.54 (t, *J* = 3.4 Hz, 4 H); ^13^C NMR (75 MHz, CDCl_3_) δ 213.8, 77.1, 76.7, 61.4, 22.9.

(ethoxy(thiocarbonyl))methyldisulfane (**3b**): pale oil (53%); TLC (SiO_2_) *R*_f_ 0.78 (hexanes:EtOAc 9:1); ^1^H NMR (300 MHz, CDCl_3_) δ 4.74 (m, 2 H), 2.53 (s, 3 H), 1.49 (tt, *J* = 7.5, 4.2 Hz, 3 H); ^13^C NMR (75 MHz, CDCl_3_) δ 212.9, 71.4, 22.9, 13.8.

### 2.2. Antimicrobial Studies

Growth media and antifungal agents were obtained from commercial vendors and used without further purification. Powder Gibco Roswell Park Memorial Institute (RPMI) 1640 medium with L-glutamine and phenol red (ref. 31800-105) was obtained from Thermo Fisher Scientific. Amphotericin B (CAS 1397-89-3) and disulfiram (97%, CAS 97-77-8) were obtained from Alfa Aesar via Thermo Fisher Scientific. Fluconazole (≥98%, CAS 86386-73-4) and voriconazole (≥98%, CAS 137234-62-9) were obtained from TCI America via VWR International.

#### 2.2.1. MIC Determination

Susceptibility testing was performed by the microdilution assay method in 96-well plate format according to CLSI protocols [19,20]. Yeast susceptibility was evaluated in modified RPMI 1640 medium supplemented with 18 g/L glucose and 34.6 g/L morpholinepropanesulfonic acid (MOPS) that was adjusted to pH 7.0 with NaOH. The same medium was used for *A. fumigatus* without the addition of glucose. Test compounds were freshly prepared in DMSO and diluted with water to 1 mg/mL stocks containing ≤5% DMSO. Fungi were enumerated from frozen glycerol–RPMI stocks on peptone dextrose agar for 2 to 4 days at 35 °C in a water-jacketed incubator. Yeast colonies and *A. fumigatus* conidia were used to prepare 0.5 McFarland suspensions (OD_530_ 0.115 to 0.130) in sterile saline. The yeasts were further diluted in the growth medium that provided a final test inoculum of 1 to 5 × 10^3^ CFU/mL after addition to two-fold dilutions of the test agents. Likewise, *A. fumigatus* conidia were diluted in modified RMPI 1640 to final test inocula of 1 to 5 × 10^4^ CFU/mL. The plates were incubated in a water-jacketed incubator at 35 °C. The modal MICs of three independent tests are reported as the lowest drug concentration that prevented discernible growth (100% inhibition) of the *Candida* species at 24 h and at 48 h for *A. fumigatus* and *Cryptococcus neoformans*.

#### 2.2.2. Growth Studies

The effect of the (dithioperoxo)thiolates on *A. fumigatus* AR-1295 growth over time was analyzed using 990 µL of a 1 to 5 × 10^5 ^CFU/mL culture in modified RPMI 1640 (165 mM MOPS, pH 7.0) treated with the vehicle and/or test agents in 10 µL of RPMI. Cultures consisting primarily of swollen conidia and germlings were prepared from dormant conidia cultured in 1:1 vol:vol medium of Hanks’ balanced salt solution (HBSS) and fetal bovine serum (FBS) at 35 °C for 6 to 8 h. The treated cultures were dispensed in 100 µL aliquots to the wells of a 96-well microtiter plate and sealed with transparent adhesive film. The plates were placed in a Molecular Devices SpectraMax^®^ 384 plate reader (Molecular Devices, San Jose, CA, USA) with a chamber temperature set to 35 °C. The OD_530_ values were recorded hourly over a 48 h period following agitation for 10 s. Growth curves were plotted against time from the OD_530_ readings using Prism software version 10.2.3 (GraphPad Software, Inc., Boston, MA, USA).

#### 2.2.3. Checkerboard Studies

Isobologram analysis was performed with *A. fumigatus* AR-1295 using the microdilution checkerboard assay in a 4 × 5 matrix format [21]. Dormant conidia were diluted in modified RMPI 1640 to a final test inocula of 1 to 5 × 10^4^ CFU/mL. Microtiter plates containing two-fold serial dilutions of (dithioperoxo)thiolate (range 0.25–16 µg/mL) and copper (II) sulfate (range 4–16 µg/mL) or glutathione (range 1–4 µg/mL) were then inoculated with *A. fumigatus* AR-1295. The plates were placed in a water-jacketed incubator and incubated at 35 °C for 48 h. The modal OD_530_ values obtained for replicates performed on three separate trial days were plotted as heat maps using Prism software version 10.2.3.

### 2.3. Cytotoxicity Studies

#### 2.3.1. IC_50_ Determination

The in vitro cytotoxicity was determined against two human cell lines (HEK293T and MDA686TU) using the sulforhodamine B (SRB) colorimetric assay [22]. HEK293T is an immortalized human cell line derived from embryonic kidney cells and purchased from ATCC. MDA686TU is a head and neck squamous cell carcinoma cell line established from primary tongue cancer and was obtained from Dr. Peter G. Sacks at New York University College of Dentistry in 2014. The cells were enumerated in Dulbecco’s Modified Eagle Medium (DMEM) and DMEM/F12 (1:1) media supplemented with 10% fetal bovine serum (FBS), respectively. Cells were incubated in a controlled environment maintained at 37 °C with a humidified atmosphere containing 5% CO_2_ until 80% confluency was achieved. About 2.5 × 10^3^ MDA686TU cells in 100 µL DMEM were seeded in each well of the 96-well plate and incubated overnight. Similarly, 5 × 10^3^ HEK293T cells were seeded in DMEM/F12 in each well. The cultures were then treated with 15, 20, 25, 30, 35 and 40 µM of the test compounds in quadruplicate for each concentration. Cisplatin was used as a positive control in parallel plates for MDA686TU cells. After 72 h of incubation at 37 °C under 5% CO_2_ atmosphere, the cells were fixed with cold 10% trichloroacetic acid (TCA) for 1 h at 4 °C, washed five times with distilled water and allowed to dry overnight. Afterwards, the cells were stained with 50 μL of 0.4% SRB for 10 min at room temperature, washed five times with 1% acetic acid, and air-dried. Absorbance was taken at OD_492_ using a microplate reader after dissolving the bound dye with 100 µL 10 mM Tris solution (pH 10.5). After calculating the growth inhibition for each concentration, CalcuSyn Software version 2.11 was used to determine the growth curve and IC_50_.

#### 2.3.2. Hematological Analysis

Trunk blood was collected in EDTA tubes from a male Sprague Dawley rat (Hilltop Lab Animals, Scottdale, PA, USA) and used within 3 h of the study. The blood (46 µL) was dispensed in 1.5 mL centrifuge tubes containing 1.25 mM of the test agents in 4 µL of DMSO. The samples were incubated at 37 °C with gentle agitation (200 rpm). Complete blood counts were analyzed using a Hemavet 950FS (Drew Scientific, Plantation, FL, USA) after 2 h. The log_2_ fold change (log_2_FC) was calculated from the mean values in the vehicle control (DMSO) versus treatment groups. Statistical significance was assessed by Student’s *t*-test. *p* values of ≤0.05 were considered significant.

## 3. Results

### 3.1. (Dithioperoxo)thiolate Synthesis

**Figure 2:**
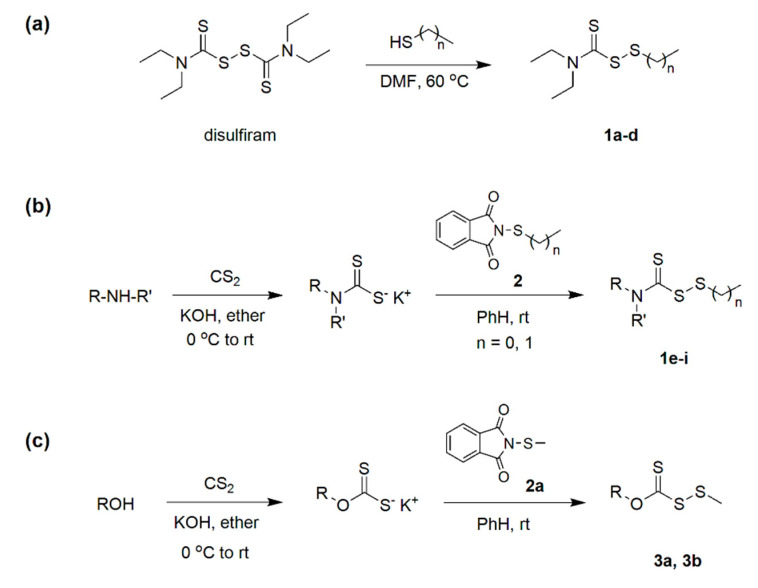
Synthesis of (dithioperoxo)thiolates. (**a**) Disulfiram-derived analogs **1a–d** were prepared from their respective mercaptans by a thiol-disulfide exchange reaction; (**b**) Carbamo(dithioperoxo)thiolates **1e–i** were prepared from their respective amines and *N*-alkylthiophthalimides (**2**); (**c**) Carbano(dithioperoxo)thiolates **3a** and **3b** were prepared from their respective alcohols and *N*-alkylthiophthalimides.

The strategies employed to synthesize the (dithioperoxo)thiolates for the investigation are depicted in Figure 2. The disulfiram-based analogs **1a–d** were prepared via a thiol-disulfide exchange reaction with their respective thiols in dimethylformamide (DMF) (Figure 2a). Following workup, silica gel chromatography afforded the pure products in modest yields of 22 to 31%. The carbamo(dithioperoxo)thiolates **1e–i** were alternatively synthesized in higher yields (42 to 63%) from potassium dithiocarbamates derived from their respective amines and carbon disulfide (Figure 2b). The intermediates were then reacted in benzene (PhH) with either *N*-methylthiophthalimide or *N*-ethylthiophthalimide (**2**) that precipitated potassium phthalimide to enable convenient product separation. Beforehand, the respective *N*-alkylthiophthalimides were prepared from phthalimide and their sulfenyl chloride precursors obtained by reacting sulfuryl chloride and methyldisulfide or ethanethiol. Lastly, the carbano(dithioperoxo)thioate analogs **3a** and **3b** were obtained in yields of 46% to 64% from their respective potassium xanthates by the same method (Figure 2c).

### 3.2. Susceptibility Testing

The MICs were determined according to protocols set forth by CLSI reference methods M27-A4 [19] and M38-A3 [20] for yeast and filamentous fungi, respectively. The fungi were treated with the test agents in MOPS-modified RPMI 1640 medium at a two-fold dilution range of 0.5 to 16 µg/mL. Per CLSI guidelines, the MIC endpoints were 24 h for *Candida* species and 48 h for *A. fumigatus* and *Cryptococcus neoformans*. Table 1 compares the modal MIC values for the disulfiram-derived (dithioperoxo)thiolates **1a–d** to disulfiram and standard antifungal drugs. The data shows a consistent SAR profile across the test panel of fluconazole-resistant *Candida* species, fluconazole-susceptible *C. neoformans*, and voriconazole-resistant *A. fumigatus* AR-0731 (L98H/TR34). Maximal antifungal activity was achieved for the *S*-ethyl derivative **1a** followed by the *S*-butyl (**1b**), *S*-hexyl (**1c**), and *S*-octyl (**1d**) analogs. Disulfiram was comparably less active against the yeast isolates than analog **1a**, while filamentous growth was observed at the 48 h endpoint for *A. fumigatus* at 16 µg/mL.

The investigation then proceeded to focus on the *S*-methyl and *S*-ethyl series of (dithioperoxo)thiolates. Table 2 compares the modal MICs for derivatives obtained from various potassium dithiocarbamates and xanthates. The data shows that only the *N*,*N*-dialkyl carbamo(dithioperoxo)thiolates **1e**, **1f**, and **1g** exhibited appreciable antifungal activity (i.e., MIC < 10 µM). Moreover, neither of the carbano(dithioperoxo)thiolates (i.e., **3a**, **3b**) inhibited the growth of *A. fumigatus* at treatments of 16 µg/mL or less (i.e., ≤95 µM). The data further shows that the MIC values for the DETC-bound *S*-methyl (**1e**) and *S*-ethyl (**1a**) (dithioperoxo)thiolates were within ±1 log_2_ dilution of each other. The same outcome was observed for the respective dimethyldithiocarbamate (DMTC) based analogs **1f** and **1g**. Altogether, the susceptibility data indicated that the *S*-methyl analogs **1e** and **1f** were more effective inhibitors of *A. fumigatus* whilst the *Candida* species appeared to be equally susceptible to the *S*-ethyl derivatives **1a**, **1g**, and **1h**.

Afterwards, the (dithioperoxo)thiolates exhibiting MICs of ≤10 µM were evaluated against a ten-member *A. fumigatus* panel comprising strains characterized for their cyp51A mutations. The results presented in Table 3 show that the *S*-methyl (**1e**, **1f**) and *S*-ethyl (**1a**, **1g**) analogs had a MIC range of 0.5 to 4 µg/mL. Although no correlations between the antifungal activity and the amino acid substitutions were evident, the *S*-methyl (dithioperoxo)thiolates **1e** and **1f** displayed the lowest variation in their modal MIC values between the mutant variants. We further compared the MICs with the primary metabolite of analogs **1a** and **1e** (i.e., DETC) that was prepared as the sodium salt by reacting 2.5 mmol each of diethylamine, carbon disulfide, and sodium hydroxide powder in 10 mL of anhydrous diethyl ether. The results in Table 3 indicate that the modal MICs of DETC•Na were at least four times higher for the *A. fumigatus* panel. However, a higher degree of variability in the MICs was observed for DETC•Na with up to ±2 log_2_ dilution difference between experiments.

### 3.3. Pharmacodynamic Studies

**Figure 3:**
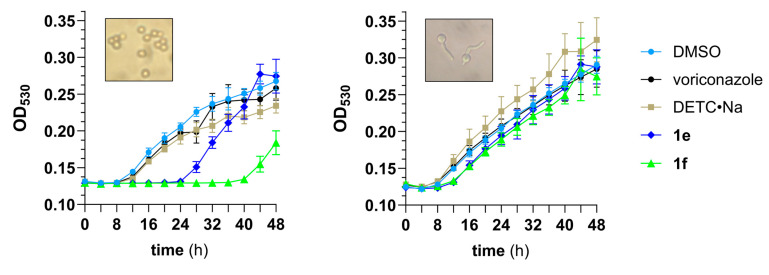
Growth response curves for a 1 to 5 × 10^5 ^CFU/mL inoculum of *A. fumigatus* AR-1295 consisting of dormant conidia (**left**) and primarily swollen conidia with emerging germ tubes (**right**) that were treated with 2 µg/mL of test agents in MOPS-modified RPMI 1640. Image magnification: 40×.

The *S*-methyl (dithioperoxo)thiolates **1e** and **1f** were selected as lead compounds to undergo further evaluation based on their optimal MIC_50_/MIC_90_ values of 2 µg/mL for the *A. fumigatus* mutant panel. A growth study was conducted using dormant conidia harvested from a six-day agar culture of *A. fumigatus* AR-1295. The results depicted in Figure 3 (left) show both analogs delayed conidial germination by at least 10 h when compared to the vehicle-treated (DMSO) control cultures. The data further indicates that DMTC analog **1f** suppressed germination and hyphal formation by up to ten hours longer than the DETC analog **1e**. By comparison, the ability of voriconazole and DETC•Na to impair filamentous growth was significantly lower than either of the (dithioperoxo)thiolates.

In the *A. fumigatus* lifecycle, the onset of conidial germination spawns isotropic swelling and emergence of germ tubes from the conidium (germlings) that continue to elongate, forming hyphae and branched hyphae called mycelium [23]. We found the same growth pattern exists in vitro for *A. fumigatus* AR-1295 when conidia from the six-day agar culture were incubated at 35 °C in Hanks’ balanced salt solution (HBSS) containing fetal bovine serum (FBS). The germlings obtained after 8 h incubation were isolated and assessed for sensitivity to (dithioperoxo)thiolates **1e** and **1f** in the study. Figure 3 (right) depicting the growth response curves indicate that the compounds impaired germling development by a similar degree. The data also indicates that voriconazole did not slow the filamentous growth of *A. fumigatus*, while treatment with DETC•Na resulted in a slightly elevated rate of hyphal formation.

**Figure 4:**
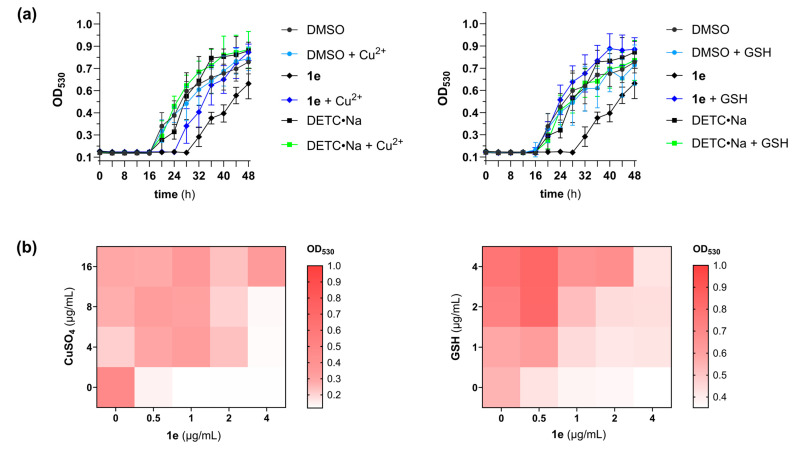
Pharmacodynamic studies on (dithioperoxo)thiolate **1e** revealed antagonistic interactions with Cu^2+^ and glutathione (GSH). (**a**) Differential growth curve analysis showed that *A. fumigatus* AR-1295 cultures containing 20 µM copper (II) sulfate or GSH were less susceptible to the (dithioperoxo)thiolate. (**b**) Heat map representing the median growth levels in the wells of checkerboard microtiter plates illustrates the antagonistic relationships of (dithioperoxo)thiolate **1e** with Cu^2+^ and GSH.

In a prior report, Shanholtzer et al. established that DETC•Na and Cu^2+^ had a synergistic interaction with up to a sixteen-fold reduction in the MICs for *Candida glabrata* and *Candida auris* [9]. The synergistic potential of the DETC-based analog **1e** and Cu^2+^ was thereby assessed by differential growth curve and isobologram (checkerboard) analyses using *A. fumigatus* AR-1295 [21,24]. Surprisingly, both experiments revealed an antagonistic relationship between Cu^2+^ and the (dithioperoxo)thiolate. Figure 4a (left) shows filamentous growth was detectable 4 h earlier for conidia co-treated with 10 µM copper (II) sulfate. A heat map comparing the growth levels in the checkboard microtiter plate wells further confirmed that Cu^2+^ decreases the antifungal activity of analog **1e** (Figure 4b, left).

As illustrated in Figure 1b, reactions between sulfhydryl-containing molecules (RSH) and (dithioperoxo)thiolate **1e** by thiol-disulfide exchange generate a mixed disulfide (i.e., RS-DETC) and DETC. The most abundant thiol that fungi use to preserve intracellular redox homeostasis and cope with oxidative stress is glutathione (GSH) [25]. It was therefore believed that GSH protects *A. fumigatus* from thiol damage by the (dithioperoxo)thiolates. To probe for antagonism by GSH, the pharmacodynamic interaction with (dithioperoxo)thiolate **1e** was examined by differential growth curve and isobologram analyses. The growth curves in Figure 4a (right) show filamentous growth was detectable 12 h earlier in the (dithioperoxo)thiolate cultures co-treated with GSH than without. The results of the checkerboard assay in Figure 4b (right) further corroborate this result of an antagonistic relationship between (dithioperoxo)thiolate **1e** and GSH.

### 3.4. Cytotoxicity Studies

The in vitro cytotoxic activities of the lead (dithioperoxo)thiolates **1e** and **1f** were evaluated with two cultured human cell lines. The analogs exhibited IC_50_ values greater than 40 µM for human embryonic kidney cells (HEK293T) using the sulforhodamine B (SRB) colorimetric assay [22]. The compounds were likewise tested on the head and neck squamous cell carcinoma cell line MDA686TU. The data indicated that the carcinoma cells were more sensitive to the respective (dithioperoxo)thiolates with IC_50_ values of 22.5 ± 5.8 and 20.8 ± 2.7 µM. Additionally, cytotoxic activities were assessed using whole blood collected from a male Sprague Dawley rat. A Hemavet analyzer was used to measure the changes in blood cell counts following 2 h incubation with compound **1e**. For the study, amphotericin B was enlisted as a comparator drug with known hemolytic activity [26]. The log_2_ fold change (log_2_FC) was calculated from the average measurements for the treated versus untreated groups. The log_2_FC and *p* values revealed that 100 µM of compound **1e** induced a moderate decrease in erythrocyte abundance (−0.29, *p* 0.15) while reductions in neutrophils and lymphocytes were not observed. By comparison, only a significant decrease in erythrocytes (−0.55, *p* 0.02) was observed with 100 µM amphotericin B and not for the other blood cell types.

## 4. Discussion

The primary objective of this research was to identify lead (dithioperoxo)thiolates as antifungal agents against *A. fumigatus*. The MIC data supported earlier findings that antimicrobial activity was contingent on the chain length of the thioalkyl substituent. Prior antimicrobial studies revealed that *C. albicans* and Gram-negative bacteria (e.g., *Acinetobacter baumannii*) were the most susceptible to DMTC and DETC derivatives bound with an *S*-ethyl substituent [13,14]. Conversely, Gram-positive bacteria, including *Staphylococcus aureus,* were more susceptible to the *S*-hexyl and *S*-octyl analogs within those series [13,14]. Our results aligned with the prior SAR trends observed with *C. albicans* and further established that the thiomethyl-bound analogs maximally suppressed the growth of fungi. It was concluded that the *S*-methyl derivatives **1e** and **1f** offered the optimal combination of potency (MIC_90_ 2 µg/mL) and lowest degree of MIC variability among the ten triazole-resistant variants in *A. fumigatus* panel (range: 1 to 2 µg/mL) for advancing as lead compounds in the investigation.

The ensuing pharmacodynamic studies revealed that compounds **1e** and **1f** inhibited the germination of dormant conidia from a triazole-resistant *A. fumigatus* mutant by 10 h or longer (Figure 3). The data further showed the period of impaired growth was significantly shorter with germinated conidia, which suggests the drugs may have limited therapeutic efficacy for progressing infections. Another notable observation was the antagonistic interaction of the DETC-based analog **1e** with Cu^2+^. This result can be partly explained by the ability of DETC to chelate heavy metals such as Cu^2+^, which is known to be toxic to *A. fumigatus* in excess [27]. Consistent with this finding, the Cu^2+^-supplemented group treated with DETC•Na was found to have accelerated growth by the 32 h time point (Figure 4a). These data provide evidence for the protective effect of DETC•Na to heavy metals in *A. fumigatus*, which was investigated as a potential chelation therapy for nickel (II) poisoning [28].

Studies on the mechanisms of antifungal action of (dithioperoxo)thiolates will be a focus of future research. Previous investigations into the antibacterial mechanisms of disulfiram and the natural thiosulfinate allicin (garlic) should allow us to glean information on the antimicrobial pharmacology in fungi. The thiol-reactive compounds [29] were shown to induce an oxidative response in *S. aureus* that includes enrichment of the biosynthetic genes for its primary antioxidant thiol, bacillithiol [12,30]. In *Escherichia coli*, allicin was shown to deplete GSH and inactivate metabolic enzymes through *S*-allylmercapto modification of cysteine residues [31]. It is therefore projected that collateral damage stemming from oxidant accumulation and deficient GSH-mediated protein repair will be correlated to the antifungal mechanism of (dithioperoxo)thiolates.

As in the case of disulfiram, the DATC metabolite is expected to play a role in the mechanism of antifungal action. Prior differential transcriptomics and bioenergetic studies implicated DETC in the antibacterial action of disulfiram as an inhibitor of oxidative phosphorylation in *S. aureus* [12]. Moreover, the studies indicated that disulfiram induces feedback inhibition of central glucose catabolism by depleting coenzyme A in *S. aureus* [12]. It is speculated that the (dithioperoxo)thiolates will have analogous antagonistic effects on fungal cellular respiration pathways and coenzyme A. It is noteworthy that cellular respiration and protein synthesis are required to break dormancy in *A. fumigatus* conidia [32]. Therefore, pharmacological studies will need to consider the effects (dithioperoxo)thiolates and their DATC metabolite on conidia germination and proliferating cells.

As with other thiol-reactive disulfides [29], the pharmacokinetics and pharmacodynamics of (dithioperoxo)thiolate-based antifungals will be influenced by their stability to the GSH levels in the blood and tissues [33]. Inhalational administration may therefore be the optimal delivery route in the treatment of pulmonary aspergillosis. Their physiochemical properties further suggest that the (dithioperoxo)thiolates will have a large volume of distribution and high tissue penetration [34]. The lipophilic attribute would be advantageous for fungal infections involving lung, bone, and brain tissues. Conversely, the compounds may not be suitable treatments for fungemia if therapeutic blood levels cannot be achieved due to hepatic metabolism and a high volume of distribution. The pharmacokinetic properties of (dithioperoxo)thiolates with respect to metabolism and distribution may thereby limit the types of infections that they can be used to treat.

## 5. Conclusions

This research established the *S*-methyl (dithioperoxo)thiolates as a lead series of compounds for antifungal drug development. It was noted, however, that the MIC values of the *S*-ethyl analogs were generally within the range of experimental error and also showed promise as a lead series. Our future studies will focus on the DETC-based analog **1e** given that the pharmacokinetic and safety parameters of disulfiram and the DETC metabolite are established [10,35].

## Figures and Tables

**Table 1:** Antifungal activity of diethylcarbamo(dithioperoxo)thioate derived from disulfiram.

Compd	n	Species ^a^|Modal MIC: µg/mL (µM)						
		*C. albicans* AR-0761 ^b^	*C. glabrata* AR-1104 ^b^	*C. krusei* AR-0397 ^b^	*C. tropicalis* AR-1098 ^b^	*C. auris* AR-1104 ^b^	*C. neoformans* NIH-306 ^c^	*A. fumigatus* AR-0731 ^c^
**1a**	1	0.5 (2)	1 (5)	0.5 (2)	1 (5)	2 (10)	1 (5)	2 (10)
**1b**	3	1 (2)	2 (8)	2 (8)	2 (8)	4 (17)	1 (4)	4 (17)
**1c**	5	1 (4)	2 (8)	2 (8)	2 (8)	4 (15)	1 (4)	8 (30)
**1d**	7	2 (7)	2 (7)	4 (17)	16 (55)	16 (55)	2 (7)	>32 (>109)
disulfiram		1 (3)	8 (27)	1 (3)	8 (27)	2 (7)	8 (27)	>16 (>54)
fluconazole		>16 (>52)	>16 (>52)	>16 (>52)	>16 (>52)	>16 (>52)	8 (23)	>16 (>52)
voriconazole		>16 (>45)	4 (12)	2 (6)	8 (23)	1 (3)	≤0.5 (≤1)	4 (12)
amphotericin B		1 (1)	0.5 (0.5)	1 (1)	1 (1)	1 (1)	0.5 (0.5)	1 (1)

^a^ Species: Candida albicans, Candida glabrata, Candida krusei, Candida tropicalis, Candida auris, Cryptococcus neoformans, Aspergillus fumigatus. ^b^ MIC endpoint: 24 h ^c^ MIC endpoint: 48 h.

**Table 2:** Antifungal activity of (dithioperoxo)thiolates derived from potassium dithiocarbamates and xanthates.

Compd	R	R’	n	Species|Modal MIC: µg/mL (µM)						
				*C. albicans* AR-0761 ^a^	*C. glabrata* AR-1104 ^a^	*C. krusei* AR-0397 ^a^	*C. tropicalis* AR-1098 ^a^	*C. auris* AR-1104 ^a^	*C. neoformans* NIH-306 ^b^	*A. fumigatus* AR-0731 ^b^
**1e**	Et	Et	0	1 (5)	0.5 (3)	1 (5)	0.5 (3)	1 (5)	2 (10)	2 (10)
**1f**	Me	Me	0	1 (6)	2 (12)	2 (12)	2 (12)	2 (12)	2 (12)	1 (6)
**1g**	Me	Me	1	0.5 (3)	1 (6)	1 (6)	1 (6)	1 (6)	1 (6)	1 (6)
**1h**	Me	Bn	1	1 (4)	0.5 (2)	0.5 (2)	2 (8)	2 (8)	0.5 (2)	4 (16)
**1i**	Me	MeO	0	1 (6)	1 (5.5)	1 (6)	2 (11)	1 (6)	4 (22)	4 (22)
**3a**	Me		0	16 (104)	8 (52)	16 (104)	16 (104)	8 (52)	8 (52)	>16 (>104)
**3b**	Et		0	16 (95)	8 (48)	16 (95)	16 (95)	8 (48)	8 (48)	>16 (>95)

^a^ MIC endpoint: 24 h ^b^ MIC endpoint: 48 h.

**Table 3:** Susceptibility of characterized isolates of voriconazole-resistant *A. fumigatus*.

Strain	Cyp51A Mutation	Compd|Modal MIC: µg/mL (µM)					
		1a	1e	1f	1g	DETC•Na ^a^	Voriconazole
AR-0732	F495I, L98H, S297T, TR34	2 (10)	2 (10)	1 (6)	2 (11)	>16 (>71)	2 (6)
AR-0733	L98H, TR34	2 (10)	2 (10)	1 (6)	1 (6)	>16 (>71)	4 (12)
AR-0734	L98H, TR34	4 (19)	2 (10)	1 (6)	4 (22)	>16 (>71)	4 (12)
AR-0735	F495I, L98H, S297T, TR34	2 (10)	2 (10)	1 (6)	2 (11)	>16 (>71)	2 (6)
AR-1283	M220V	2 (10)	2 (10)	2 (12)	1 (6)	>16 (>71)	1 (3)
AR-1292	M220K	2 (10)	2 (10)	2 (12)	1 (6)	>16 (>71)	2 (6)
AR-1293	G54R	1 (5)	1 (5)	2 (12)	1 (6)	>16 (>71)	2 (6)
AR-1294	T289A, TR46, Y121	1 (5)	1 (5)	2 (12)	2 (11)	≥16 (≥71)	>16 (>45)
AR-1295	T289A, TR46, Y121	4 (19)	2 (10)	2 (12)	4 (22)	≥16 (≥71)	>16 (>45)
AR-1296	T289A, TR46, Y121	1 (5)	1 (5)	2 (12)	4 (22)	>16 (>71)	>16 (>45)

^a^ sodium diethyldithiocarbamate.

## Data Availability

The original contributions are included in the article; further inquiries can be directed to the corresponding author.
